# Self-efficacy, reflection, and resilience as predictors of work engagement among English teachers

**DOI:** 10.3389/fpsyg.2023.1160681

**Published:** 2023-05-12

**Authors:** Qingzhi Heng, Lina Chu

**Affiliations:** Department of Basic Education, Chongqing Creation Vocational College, Yongchuan, Chongqing, China

**Keywords:** work engagement, self-efficacy, reflection, resilience, EFL teachers, teacher education programs

## Abstract

**Introduction:**

Given the significant role of work engagement of teachers in educational contexts, some scholarly attention has been directed to exploring the predictors of this construct. Against this backdrop, this study aimed to investigate the predictors of teacher work engagement among Chinese English as a foreign language (EFL) teachers by testing a model that includes teacher self-efficacy, teacher reflection, and teacher resilience.

**Methods:**

To achieve this goal, 512 EFL teachers were invited to participate in an online survey, which consisted of four questionnaires. The construct validity of the measures was confirmed through confirmatory factor analysis. Then, structural equation modeling was utilized to examine the relationships between the variables.

**Results:**

The findings indicated that teacher self-efficacy, teacher reflection, and teacher resilience were direct predictors of work engagement, and teacher self-efficacy had an indirect effect on work engagement via teacher reflection and resilience. Similarly, teacher reflection also had an indirect impact on work engagement through teacher resilience.

**Discussion:**

These results have important implications for teacher education programs. The significance of these predictors of work engagement among EFL teachers highlights the importance of fostering self-efficacy, reflection, and resilience among teachers in order to promote their work engagement. Further research can explore ways to enhance these predictors through training and support programs for teachers.

## Introduction

Teacher work engagement has been identified as a crucial aspect in determining the success of educational outcomes (Timms and Brough, 2013; Perera et al., 2018). Given its significance, there is growing interest in examining the factors that predict teacher work engagement (Eldor and Shoshani, 2016; Greenier et al., 2021). Work engagement is described as a state characterized by passion, energy, and motivation toward work and has been linked to physical and psychological wellbeing at work as well as a pleasing and satisfying state of mind (Rothmann, 2008; Bakker et al., 2011; Greenier et al., 2021). This concept has gained recognition as a key indicator in evaluating the quality of teachers’ professional lives and its influence on various work-related consequences including instruction quality, problem-solving skills, organizational commitment, and work satisfaction (Field and Buitendach, 2012). Furthermore, studies have found that engagement is positively associated with teacher efficacy and can act as a bridge between social support and efficacy (Bresó et al., 2011; Høigaard et al., 2012; Han and Wang, 2021; Namaziandost et al., 2022). According to Schaufeli et al. (2002), individuals who are dedicated and passionate about their work tend to be more engaged in their profession. Cardwell (2011) also emphasized that heightened work engagement can positively impact instruction effectiveness. As a result, there has been a growing body of research exploring the factors that impact EFL/ESL teachers’ work engagement (Burić and Macuka, 2018; Greenier et al., 2021; Xie, 2021).

Self-efficacy, as the second construct under research in the study, refers to a person’s beliefs and perceptions about their capability to attain specific outcomes in particular settings (Bandura, 1997). Bandura (1993) proposed that a person’s view of their abilities influences their confidence or proficiency in handling demanding and challenging circumstances, which in turn influences their psychological wellbeing. Specifically, teacher efficacy is concerned with a teacher’s self-evaluation of their instructional capabilities in achieving desired outcomes in an educational setting (Zee et al., 2018; Thompson and Dooley, 2019; Liu et al., 2021; Samfira and Paloş, 2021). It is acknowledged as a crucial factor that affects both students’ motivational beliefs and the quality of a teacher’s instruction (Xiyun et al., 2022). Self-efficacious teachers are more inclined to utilize advanced instructional methods and persistently work with struggling students (Gibson and Dembo, 1984). According to Bandura (1997), self-efficacy stems from four main sources: verbal persuasion, vicarious experience, mastery experience, and emotional arousal. Among these sources, mastery experience is considered the most crucial in forming self-efficacy as it involves a teacher’s previous knowledge of their students’ success, which increases their sense of efficacy, and their students’ experience of failure, which decreases their sense of efficacy.

Resilience is another critical factor that can lead to greater teacher engagement in their careers, as indicated by previous studies (Wang et al., 2022). According to Mansfield et al. (2016), resilience is defined as a personal attribute that allows teachers to effectively cope with the challenges and difficulties of teaching rather than simply endure them. Resilient instructors are argued to be more motivated, dedicated to professional development, and focused on improving their instruction, thus serving as a “quality retention” factor (Day and Gu, 2010; Zhang, 2021). These teachers possess the requisite competencies to succeed in difficult situations, excel at instructional leadership, have positive relationships with students, feel satisfied, are dedicated to their profession, and derive personal enjoyment and fulfillment from their work (Howard and Johnson, 2004; Polat and İskender, 2018; Chu and Liu, 2022; Liu and Chu, 2022). Moreover, students of resilient teachers are more likely to achieve their desired learning outcomes (Day and Gu, 2014; Derakhshan et al., 2022a). In addition to self-efficacy and resilience, teacher reflection has proved to play a significant role in a teacher’s professional growth, wellbeing, and effectiveness (Wright, 2010). Reflection allows teachers to better understand themselves and their practice and serves as a means of knowledge generation based on their experiences. Farrell (2019) emphasizes the importance of reflection in second language teacher education programs as a means of bridging the gap between theoretical abstractions and practical applications. By reflecting on their teaching practices, teachers can gain a clearer understanding of situational factors in the classroom and improve their awareness of language instruction pragmatics and the ability to apply theoretical concepts in practical settings (Wallace and Bau, 1991; Griffiths, 2000).

Although several investigations have been carried out on various factors influencing teachers’ work engagement (e.g., Hultell and Gustavsson, 2011; Burić and Macuka, 2018; Greenier et al., 2021; Xie, 2021), it is still in its infancy and less researched especially in EFL context. Both researchers and educators should devote more attention to teacher factors such as self-efficacy, reflection, and resilience since these constructs can influence teacher work engagement. Also, to our best knowledge, no study has ever probed the concurrent antecedents of teacher self-efficacy, teacher reflection, and teacher resilience on impacting teachers’ work engagement. As such, the current study tried to delve into the role of teacher self-efficacy, teacher reflection, resilience, in affecting work engagement among EFL teachers. This study aims to contribute to the existing body of knowledge by providing new insights into the predictors of teacher work engagement in the Chinese EFL context and has implications for teacher education programs.

## Literature review

### Work engagement

Work engagement has gained significant attention in various fields in recent years, and there is a growing body of literature exploring its definition, antecedents, and outcomes (Liu et al., 2020; Han and Wang, 2021; Oberländer and Bipp, 2022). Schaufeli et al. (2002) introduced the most accepted and widespread definition of work engagement, which includes three components: absorption, vigor, and dedication. Work engagement is a positive mindset that demonstrates one’s professional career, achievement, fulfillment, and efficiency (Schaufeli and Bakker, 2004; Field and Buitendach, 2012). It is not a temporary mental state but rather a more ubiquitous and lasting cognitive state that is unrelated to a single event, individual, activity, or item (Schaufeli et al., 2002; Azari Noughabi et al., 2022). This positive work-related variable is based on the philosophy of work engagement, which emphasizes the role of passion, energy, and personal satisfaction in propelling workers ahead in their job performance (Han and Wang, 2021). This construct developed from burnout research, with the goal of focusing on employees’ wellbeing and strategies to improve it rather than their level of burnout (Zeng et al., 2019; Fathi et al., 2021).

Recent research has shown that work engagement has an inverse relation with burnout and employee desire to quit (Schaufeli and Bakker, 2004; Faskhodi and Siyyari, 2018; Ahmad et al., 2021; Juliana et al., 2021; Dong and Xu, 2022) and it varies from workaholism since engagement is a positive attribute that provides beneficial results, whereas workaholism causes more harm than good and leads to burnout (Han and Wang, 2021). The literature suggests that work engagement is associated with positive feelings toward teaching (Zeng et al., 2019) and can lead to a decline in job burnout (Juliana et al., 2021). Studies exploring teacher engagement have also been done from the viewpoints of gender, teaching status, and teaching experience (Faskhodi and Siyyari, 2018; Topchyan and Woehler, 2021). In addition, perceived learner engagement and motivation are key factors in determining teacher engagement, especially in the context of online learning (Obrad and Circa, 2021). Further, engaged teachers in FL/L2 environments are more energetic, devote a significant amount of their cognitive repertoire to their work, and maintain their resilience despite obstacles (Brackett et al., 2010; Burić and Macuka, 2018; Greenier et al., 2021; Xiao et al., 2022; Derakhshan et al., 2022b). Psychological wellbeing, emotion regulation, resilience, and emotional intelligence have been identified as significant factors contributing to work engagement among teachers (Butakor et al., 2021; Greenier et al., 2021; Xie, 2021). Therefore, understanding the antecedents and outcomes of work engagement is crucial for promoting teacher wellbeing and improving job performance. Overall, a critical review of the literature suggests that work engagement is an important construct that has gained significant attention in various fields in recent years. While work engagement is associated with positive outcomes such as employee wellbeing and job performance, workaholism is associated with negative outcomes such as burnout. However, there is a need for further research to identify gaps in the literature and develop effective interventions to promote work engagement among teachers.

### Teacher self-efficacy

The concept of self-efficacy, or an individual’s belief in their ability to perform a specific task effectively, has significant implications for how people approach challenges, handle stress, and solve problems (Bandura, 1997, 2011). In the realm of education, teacher self-efficacy specifically refers to a teacher’s confidence in their ability to carry out teaching duties to a particular standard under specific circumstances (Skaalvik and Skaalvik, 2014), as outlined by Bandura (1997) social cognitive theory. Numerous studies have investigated the relationship between teacher self-efficacy and other constructs, including job satisfaction, work engagement, and organizational commitment (e.g., Skaalvik and Skaalvik, 2014; Minghui et al., 2018; Granziera and Perera, 2019; Demir, 2020; Doo et al., 2020; Han and Wang, 2021). The results indicate that teachers with greater self-efficacy tend to have higher job satisfaction, less emotional exhaustion, and lower levels of job burnout (e.g., Skaalvik and Skaalvik, 2014; Fathi et al., 2021). They are also better able to manage student behavior and collaborate effectively with colleagues to achieve common educational goals (Tsouloupas et al., 2010; Goddard and Kim, 2018; Poulou et al., 2019; Lazarides et al., 2020).

Research suggests that effective teachers foster a high-quality learning context by designing lessons that challenge students’ abilities, by handling student misbehavior skillfully, and by making an effort to engage students meaningfully (Tschannen-Moran and Barr, 2004; Tsouloupas et al., 2010). It is generally accepted that teachers who have higher levels of self-efficacy establish the atmosphere for developing stronger bonds with their students and interacting in ways that support behavioral operating in students (Hamre et al., 2008; Poulou, 2017). In another study, Burić and Macuka (2018) used a sample of Croatian instructors to demonstrate that teachers with high self-efficacy revealed greater engagement in their jobs, more confidence, love, and excitement, and less exhaustion, despair, and frustration toward their students. Language teachers’ self-efficacy is a growing area of study that has been assessed in a few East Asian settings (e.g., Phan and Locke, 2015). Furthermore, Hoang and Wyatt (2021) emphasized the critical influence of culture and context in forming the self-efficacy perceptions, instructional strategies, classroom management, and student misbehavior management of Vietnamese pre-service teachers. Additionally, self-efficacy was the best predictor of job performance among EFL teachers out of all the variables used by Soodmand Afshar and Moradifar (2021), including institutional identity, critical cultural awareness, reflective teaching, and self-efficacy. In addition, the results of the study by Greenier et al. (2021) revealed a negative connection between teacher self-efficacy and burnout. Teachers’ perceptions of their unique teaching context, the requirements of their instructional practices, and evaluations of the support and resources that are available to them all have a significant impact on how effective they are as teachers (Bandura, 1997).

According to Tschannen-Moran and Hoy (2001), teachers with higher levels of teacher self-efficacy motivate and encourage their students to succeed more than teachers with lower levels of self-efficacy. They also tend to use more constructive feedback with students who consistently make mistakes. Hajovsky et al. (2020) also found that across all grade levels, teachers with higher self-efficacy beliefs usually have higher levels of nearness and lower levels of conflict with their pupils. They thought that teachers who felt confident in their abilities to instruct and control classroom behavior were more likely to engage in behaviors that helped them build dependable relationships with their students. According to other studies (Federici and Skaalvik, 2011; Yakın and Erdil, 2012), self-efficacy has an impact on internal motivation, job satisfaction, and engagement. The more self-efficacy instructors have, the more it aids in keeping them aware in an organized working state. According to other researchers (e.g., Llorens et al., 2007), self-efficacy is an important predictor variable affecting employees’ work engagement. Simbula et al. (2011) conducted a research on teachers’ work engagement and self-efficacy using Italian teachers as the research subjects. The study demonstrated a strong relationship between teachers’ self-efficacy and work engagement. Furthermore, instructors with high self-efficacy may put in more effort and have superior organizational and planning abilities (Pajares, 1992).

In another investigation, Safari et al. (2020) discovered that EFL teacher self-efficacy is a good predictor of professional growth. It is worth noting that self-efficacy outperformed reflective thinking and work satisfaction in predicting professional progress. Furthermore, teacher self-efficacy was found to be adversely related to burnout. Von Muenchhausen et al. (2021) discovered that teacher self-efficacy and mental health were substantially and modestly connected when they examined the relationship between mental health and teacher self-efficacy among 742 instructors. Besides, positive emotions and work-related psychological barriers were associated with teacher self-efficacy. Similarly, the greater the development in life satisfaction and distancing ability, the better the teacher’s self-efficacy, and lower social support experience was associated with lower teacher self-efficacy. Furthermore, Ventura et al. (2015) found that professional self-efficacy was a strong predictor of teachers’ psychosocial wellbeing, which is technically referred to as burnout. It was also found that both burnout and engagement were significantly correlated with professional self-efficacy. Specifically, professional self-efficacy was positively correlated with engagement and self-efficacy was inversely related to burnout. In another study, Høigaard et al. (2012) discovered that engagement and efficacy perceptions are correlated with job satisfaction and inversely associated with burnout and the intention to quit among newly qualified teachers in Norway.

Burić et al. (2022) also conducted a three-wave longitudinal study among 3,010 teachers and found that stable parts of teacher self-efficacy (TSE) and work engagement have a positive correlation, TSE is positively associated with work engagement at a given time point, and work engagement has spill-over effects on TSE, but there is no reciprocal relationship. In another study, Gratacós et al. (2021) conducted a study with Spanish beginning teachers and found a strong positive correlation between transformational leadership (TR) and self-perceived efficacy, and the motivational and social dimensions of TR could be a determining factor in enhancing the adaptative skills of beginning teachers, leading to self-efficacy. Taken together, as reviewed above, teacher self-efficacy has been widely researched and has been shown to have a positive relationship with various constructs such as job satisfaction, work engagement, and organizational commitment. Research has also documented that self-efficacy is an important predictor of job performance, internal motivation, and professional growth and is also negatively correlated with burnout. While the existing literature provides an extensive overview of the studies related to teacher self-efficacy and its relationship with work engagement, the literature has yet to address further investigation of self-efficacy in language teachers. Additionally, the existing bulk of the literature does not explore the potential factors that may moderate the relationship between teacher self-efficacy and work engagement. Therefore, future research may benefit from addressing these gaps to better understand the complex relationship between teacher self-efficacy and work engagement.

### Teacher reflection

Teacher reflection is a critical component of professional growth, efficiency, and wellbeing for teachers (Akbari et al., 2010; Aleandri and Russo, 2015). Reflective teachers assess their teaching methods and adapt them as needed to enhance learning quality (Xu et al., 2015; Han and Wang, 2021). In second language (L2) teacher education, which places a strong emphasis on producing high-quality teachers, teacher reflection is crucial for bridging the gap between theory and practice (Hua, 2008; Farrell and Kennedy, 2019). Dewey (1933) and Schön (1983) were the first to use the term “reflection” to describe systematic, thoughtful, and purposeful actions that followed logical reasoning. Schön (1983) further developed the concept by defining three components: reflection-in-action, reflection-on-action, and reflection-for-action, all of which are intended to enhance learning and teaching. Reflection-in-action takes place as part of an instructional practice, reflection-on-action takes place as a consequence of the practice, and reflection-for-action improves or changes future actions.

Reflective teaching is a dynamic process that evolves over time with proper training (Yalcin Arslan, 2019). As a result, there is a burgeoning interest in the educational community to enhance teachers’ ability to reflect, as it has been linked to various factors, including engagement, autonomy, burnout, self-efficacy, perfectionism, and teaching-learning beliefs (Moradkhani et al., 2017; Korucu-Kis and Demir, 2019; Loan, 2019; Xiaojing et al., 2022). Akbari et al. (2010) proposed a framework for teacher reflection that classifies it into five categories: affective, practical, cognitive, meta-cognitive, and critical. The practical category involves the techniques and tools that teachers use in reflective teaching, the affective component relates to teachers’ reflection on their students’ difficulties, the cognitive aspect deals with teachers’ efforts at professional growth, and the meta-cognitive component involves teachers’ evaluation of their activities. The critical component encompasses teachers’ perspectives on the sociopolitical impact of their actions.

Researchers have demonstrated the significance of reflective teaching in various contexts (Cimermanová, 2013; Cabaroglu, 2014; Košir et al., 2015; Moradkhani et al., 2017; Motallebzadeh et al., 2018; Shirazizadeh and Moradkhani, 2018; Walshe and Driver, 2019; Gorski and Dalton, 2020). As far as L2 context is concerned, Moradkhani et al. (2017) conducted a study to investigate the relationship between EFL teachers’ reflection and their self-efficacy. The findings showed that all elements of reflection, except for critical reflection, were significantly linked to self-efficacy. The meta-cognitive component was found to be the only indicator of self-efficacy. Qualitative data analysis revealed that the components of reflection improved self-efficacy through four primary sources: mastery experience, vicarious experience, verbal persuasion, and physiological/emotional arousal. Košir et al. (2015) also found that rumination was a significant contributor to burnout and stress among schoolteachers, while reflection acted as a mediator between teachers’ career qualities and stress. Cimermanová (2013) suggested that self-reflection was effective in reducing burnout among school and university teachers. In another study, Shirazizadeh and Moradkhani (2018) investigated the relationship between reflective practices of EFL teachers and burnout. The results showed that participation in reflective practice was negatively correlated with burnout, indicating that engaging in reflective practices was linked to a reduction in burnout. Likewise, Motallebzadeh et al. (2018) also found a positive relationship between reflection and teaching efficacy among EFL instructors. Cabaroglu (2014) evaluated the impact of action research on the self-efficacy beliefs of pre-service EFL teachers and found that it contributed to the improvement of their self-efficacy, self-awareness, problem-solving skills, and learning autonomy. These studies might suggest that reflective practices, such as action research, can contribute to the improvement of EFL teachers’ self-efficacy and reduce burnout.

Overall, although the literature review provides a comprehensive understanding of the relationship between teacher reflection and various aspects of teaching, including burnout, self-efficacy, and teaching efficacy, there appears to be a gap in terms of investigating the role of resilience in this relationship. Resilience is an important aspect of teacher wellbeing, particularly in challenging educational contexts, and can potentially impact teacher engagement (Chen and Chi-Kin Lee, 2022). Therefore, the current study aims to fill this gap by examining the relationship between teacher self-efficacy, reflection, resilience, and work engagement among English language teachers.

### Teacher resilience

Teacher resilience is the ability to adapt to a variety of settings and strengthen abilities in overcoming difficulties (Bobek, 2002; Mansfield et al., 2016; Liu and Han, 2022). Resilient teachers consistently demonstrate agency, moral purpose, strong support groups, a sense of achievement, and enthusiasm (Stanford, 2001; Howard and Johnson, 2004). Resilience is a dynamic process that is impacted by various psychological, biochemical, and environmental-contextual processes in addition to individual features, familial factors, and the social context (Kostoulas and Lämmerer, 2018) and it occurs when people integrate their personal and contextual resources and utilize effective techniques to overcome problems and preserve their wellbeing (Liu et al., 2021; Zhang, 2021). Resilient teachers enjoy greater job satisfaction, positive self-belief, general wellbeing, and a greater sense of commitment to their fields (Richards et al., 2016). Day (2008) argued that resilient teachers are those who demonstrate the ability to succeed in difficult situations, are excellent at classroom management, and develop strong bonds with their students. Chen and Chi-Kin Lee (2022) found that decision latitude and school support influenced the professional and emotional dimensions of teacher resilience, respectively, and predicted teacher wellbeing. The motivational and social dimensions of teacher resilience positively impacted teacher job performance and suggested that teacher resilience can mitigate negative job demands and enhance positive job resources, leading to improved wellbeing and performance. Van Wingerden and Poell (2019) found that work engagement and job crafting completely mediated the relationship between meaningful work and teacher resilience, emphasizing the significance of fostering teachers’ resilience to maintain enthusiasm in their demanding but meaningful profession. Yada et al. (2021) found a three-factor structure for self-efficacy, which was highly correlated with resilience. These studies suggest that promoting teacher resilience is crucial for maintaining teacher wellbeing, job satisfaction, and performance, and that it is influenced by various factors in the individual, organizational, and contextual levels. Further research is needed to identify effective interventions and strategies for fostering teacher resilience.

Also, some recent studies carried out by Liu et al. (2021) and Chu and Liu (2022) investigated teacher self-efficacy and teacher resilience among Chinese EFL teachers. Liu et al. (2021) found that EFL teachers had moderate-to-high levels of self-efficacy in general, with higher levels of technological self-efficacy than instructional self-efficacy. Chu and Liu (2022) reported that resilience in Chinese senior high school EFL teachers was moderate to high, with a tri-factorial structure of tenacity, optimism, and coping style. Personal and contextual factors influencing teacher resilience were discussed. Liu and Chu (2022) further explored EFL teacher resilience and discovered a tri-factorial structure of TR involving tenacity, optimism, and coping style. The study also highlighted the moderate-to-high levels of EFL teacher resilience and offered implications for sustaining and developing EFL teacher resilience. These findings offer suggestions for enhancing teacher self-efficacy and developing teacher resilience in the EFL context.

While there is considerable research on teacher resilience, there remains a gap in understanding how teacher self-efficacy and reflection contribute to teachers’ work engagement. While some studies have explored the impact of teacher resilience on wellbeing and job performance, the present study aims to investigate the interplay between teacher self-efficacy, teacher reflection, and resilience as predictors of teachers’ work engagement among English language teachers. This study will contribute to the literature by identifying specific factors that can enhance teachers’ work engagement and by highlighting the importance of promoting these factors in teacher training and professional development programs.

## The present study

The present research aims to examine the complex relationships between teacher self-efficacy, teacher reflection, teacher resilience, and work engagement among English language teachers. The four variables are logically connected and can be put together into a model based on previous research findings (as explained in the hypotheses below). Based on prior research, the researchers hypothesize that teacher self-efficacy positively affects teacher reflection, teacher reflection positively affects teacher resilience, and both teacher self-efficacy and teacher reflection positively affect teacher work engagement. Additionally, the researchers suggest that teacher resilience positively affects teacher work engagement. By examining these predictors of work engagement, the study aims to identify key factors that can help promote teacher engagement and wellbeing in EFL educational contexts. By exploring the relationships among the four constructs, the present research offers a more thorough understanding of the constructs that influence teachers’ work engagement. This could be beneficial in terms of providing insights into how to support teachers in their professional development, and to enhance the effectiveness of their instruction in EFL contexts.

**Figure 1:**
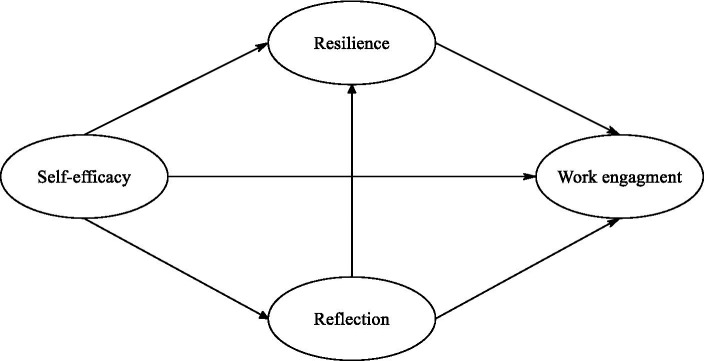
The hypothesized model.

Against this backdrop, this study set out to test a model of teacher work engagement and its predictors (see the hypothesized model in Figure 1), including teacher self-efficacy, teacher reflection, and teacher resilience among Chinese EFL teachers. The following hypotheses were also developed to guide the study:

*H1*: Teacher self-efficacy positively affects teacher reflection. This hypothesis is based on the premise that teachers who have a high level of self-efficacy are more likely to reflect on their practices, as they believe in their abilities to influence the outcomes of their teaching (Bandura, 1977). Empirical studies have consistently shown that teacher self-efficacy is positively related to various aspects of teacher reflection (Babaei and Abednia, 2016; Moradkhani et al., 2017).

*H2*: Teacher reflection positively affects teacher resilience. This hypothesis builds upon the notion that reflective practices can help teachers deal with stress and uncertainty in their work, leading to their heightened resilience (Schön, 1983; Ebersöhn, 2014; Chen and Chi-Kin Lee, 2022). By reflecting on their experiences, teachers are able to gain a deeper understanding of the challenges they face, and identify potential solutions to mitigate stress and increase resilience (Zeichner and Liston, 2013; Zulfikar and Mujiburrahman, 2018).

*H3*: Teacher self-efficacy positively affects teacher resilience. Following social cognitive theory of self-efficacy (Bandura, 1997, 2011), which posits that self-efficacy plays a crucial role in individuals’ ability to cope with adverse circumstances, it is hypothesized that teachers with high levels of self-efficacy are better equipped to bounce back from setbacks and remain engaged in their work (Yada et al., 2021).

*H4*: Teacher self-efficacy affects teacher work engagement. Based on the theory of work engagement (Schaufeli et al., 2006), which suggests that work engagement is positively related to various antecedent variables, including self-efficacy, teachers who feel confident in their abilities are more likely to be engaged in their work and motivated to continue their professional development (Simbula et al., 2011; Burić and Macuka, 2018).

*H5*: Teacher reflection affects teacher work engagement. This hypothesis builds upon the notion that reflection contributes to teacher development, which in turn leads to increased work engagement (Schön, 1983). Teachers who reflect on their practices are better able to identify areas for improvement and adopt new strategies, which enhances their sense of engagement and motivation (Han and Wang, 2021).

*H6*: Teacher resilience affects teacher work engagement. In light of some previous studies (e.g., Van Wingerden and Poell, 2019; Xie, 2021), it is suggested that individuals who are resilient are better able to manage stress and remain engaged in their work. Teachers with high levels of resilience are less likely to be affected by stress and burnout, and are therefore more likely to remain engaged in their work (Perera et al., 2018). In light of these hypotheses, the present study aims to explore the relationships among the four latent variables, and to identify the key predictors of work engagement among Chinese EFL teachers.

## Method

### Participants

The participants in this study were a convenient sample of 526 English teachers working in mainland China. The sample was composed of 218 male English teachers (41.2%) and 308 female English teachers (58.8%). The average age of the participants was 33.16 years (*SD* = 6.38 years), and the mean number of years of experience as English teachers was 10.18 (*SD* = 7.02). The participants were selected from various English language schools in mainland China, with the aim of obtaining a diverse sample of English teachers to participate in the study. The data gathered from these participants provided valuable insights into the experiences and perspectives of English teachers working in mainland China. Due to the outbreak of COVID-19 in 2020, online teaching became the predominant mode of instruction for a large number of schools in mainland China. As a result, the majority of participants in this study (84.3%) reported that they had been involved in online teaching during the past 2 years. The remaining participants reported that they had primarily engaged in offline teaching during the same period. However, it is worth noting that some of the participants who reported primarily engaging in offline teaching had also engaged in online teaching to some extent during the pandemic. Thus, the sample included a mixture of teachers who had taught exclusively online, in a mixed model (online and offline), or exclusively offline.

### Instruments

#### Teacher self-efficacy scale

The level of teacher self-efficacy was evaluated through the use of the short version of the Teachers’ Sense of Self-Efficacy Scale (TSES) created by Tschannen-Moran and Hoy (2001). The TSES aims to assess the teacher’s self-efficacy in three distinct areas: instructional strategies, student engagement, and classroom management. The scale is comprised of 12 questions, which are rated on a nine-point Likert scale that ranges from 1 (Nothing) to 9 (A great deal). Each of the self-efficacy dimensions is represented by four items.

#### Utrecht work engagement scale (UWES)

The Utrecht Work Engagement Scale which was validated by Schaufeli et al. (2002) was used to measure teachers’ work engagement. The scale is comprised of 17 questions rated on a 7-point Likert scale, and measures three sub-domains: vigor, dedication, and absorption. Vigor reflects a person’s energy, resilience, and determination even in the face of challenges. Dedication refers to a feeling of inspiration and excitement. Absorption signifies a full focus on teaching activities.

#### Teacher reflection scale

The English Language Teaching Reflection Inventory (Akbari et al., 2010) was used to measure teacher reflection. It consists of 29 items that assess teachers’ views on five different areas: practical, cognitive, affective, metacognitive, and critical. The assessment uses a 5-point Likert scale, ranging from 1 (never) to 5 (always). The overall score of the inventory reflects the level of teacher reflection across all five dimensions.

#### Teacher resilience scale

The study utilized the 10-item Connor-Davidson Resilience Scale (CD-RISC) developed by Campbell-Sills and Stein (2007). This shortened version was based on the original 25-item CD-RISC, which was developed and validated by Connor and Davidson (2003) as a multidimensional measurement tool for resilience. The questionnaire consists of Likert-type items, with responses ranging from 0 (not true at all) to 4 (true nearly all the time).

### Procedure

This cross-sectional study was carried out in mainland China, targeting teachers working in English language schools. Participants were informed of the details and purpose of the study and were given a battery of questionnaires, including those assessing socio-demographic information and the four constructs being investigated: teacher self-efficacy, work engagement, teacher reflection, and resilience. Online surveys were administered in March and April 2022 using a popular online survey platform called Questionnaire Star. The survey was sent to a sample of EFL teachers in Chinese language schools who voluntarily agreed to participate in the study. To ensure a diverse sample, the participants were also encouraged to share the survey with their colleagues via commonly used social media apps, such as WeChat and QQ, as well as through emails. Confidentiality was ensured for the questionnaire data to preserve the anonymity of the teachers. Participation was voluntary and without monetary compensation, and all participants provided written informed consent to participate in the study. The data collection process lasted approximately 2 months.

### Data analysis

The statistical analysis was performed using the SPSS 22.0 for descriptive statistics and data input and the AMOS 20 software for Confirmatory Factor Analyses (CFAs) and Structural Equation Modeling (SEM). The data was screened to evaluate missing data, outliers, and normality. The expectation–maximization algorithm was used to address missing data, in which missing scores were substituted with a predictive distribution (Kline, 2011). Both univariate and multivariate outliers were examined using standard scores and Mahalanobis D^2^, respectively. A case was considered a multivariate outlier if its D^2^ probability was 0.001 or lower, and outliers were subsequently removed, resulting in 512 valid cases for SEM analysis. The skewness and kurtosis values were within the acceptable range of −1 to +1, which indicated a normal distribution of the data (Tabachnick et al., 2013). The descriptive statistics and correlation matrix for all variables are presented in Table 1.

**Table 1:** Descriptive statistics and correlations.

	*M* (*SD*)	1	2	3	4
(1) Self-efficacy	4.03 (0.93)	1.00			
(2) Reflection	3.95 (1.08)	0.26*	1.00		
(3) Resilience	3.59 (0.96)	0.32*	0.35*	1.00	
(4) Work engagement	4.12 (1.12)	0.53**	0.41**	0.34*	1.00

*p < 0.05, **p < 0.01. Asterisks denote statistical significance based on a two-tailed test (Cumming, 2014).

CFA was applied to assess the validity of the four latent variables in the study. To evaluate the goodness of the measurement models, various fit indices were used, including χ^2^/df, goodness-of-fit index (GFI), Tucker-Lewis index (TLI), comparative fit index (CFI), and root mean square error of approximation (RMSEA) (Kline, 2011). The validity of the models was evaluated based on established criteria, with a χ^2^/df value of less than 3 considered acceptable (Tseng and Schmitt, 2008) and fit indices of ≥0.90 considered acceptable (Hu and Bentler, 1999). For RMSEA, values of ≤0.06 were considered indicative of good fit and values of ≤0.08 were considered fair fit (Hu and Bentler, 1999; Kline, 2011). Due to low loadings, modifications were made to the models by removing some items from the questionnaires of teacher reflection, work engagement, and teacher self-efficacy. After the revisions, all models showed acceptable fit to the data as shown in Table 2. The reliability of the questionnaires was confirmed by their coefficient alphas, which were all higher than 0.70 (Hair et al., 2010; Table 2).

**Table 2:** Measurement model of the latent variables.

	χ^2^	Df	χ^2^/df	CFI	TLI	RMSEA	Cronbach’s α
Self-efficacy	202.35	99	2.04	0.92	0.91	0.06	0.83
Reflection	136.71	69	1.98	0.95	0.94	0.05	0.79
Resilience	84.39	46	1.83	0.98	0.97	0.03	0.86
Work engagement	68.09	33	2.06	0.93	0.93	0.06	0.80

### Model testing

**Figure 2:**
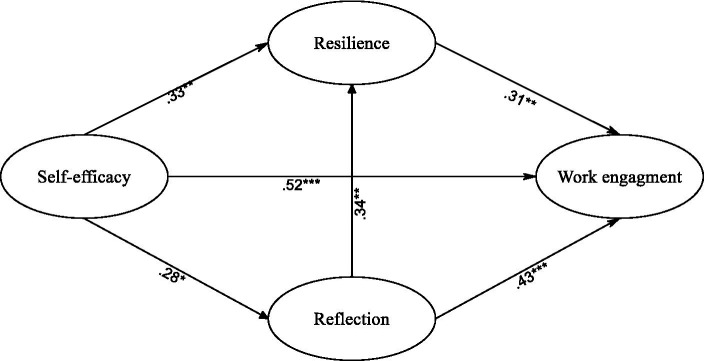
The final model. *p < 0.05. ** *p* < 0.01. ***p < 0.001. Asterisks denote statistical significance based on a two-tailed test (Cumming, 2014).

The proposed model was analyzed using AMOS 23.0, which utilized the maximum likelihood procedure and variance–covariance matrices. However, the initial assessment of the model revealed that it did not fit the data well, as indicated by the low values of GFI, TLI, and CFI, which were all below the recommended threshold of 0.90 (Hu and Bentler, 1999). To improve the model fit, modifications were made, leading to the final model depicted in Figure 2. The modifications resulted in substantial improvement in the goodness-of-fit of the model, as evidenced by the values in Table 3. Additionally, the regression coefficients for the paths in the final model were found to be significant at the level of 0.05 or lower (*p* < 0.05) (Hair et al., 2010).

**Table 3:** Fit indices for the initial and revised models.

	χ^2^	Df	χ^2^/df	CFI	TLI	RMSEA
Initial model	594.68	255	2.33	0.89	0.88	0.08
Revised model	476.80	253	1.89	0.97	0.96	0.04

As can be seen in Figure 2, self-efficacy, reflection, and resilience were the three direct significant predictors of work engagement. Self-efficacy was the strongest direct predictor of work engagement (*β* = 0.52, *R*^2^ = 0.27). Reflection (*β* = 0.43, *R*^2^ = 0.18) and resilience (*β* = 0.31, *R*^2^ = 0.09) also directly predicted work engagement. Furthermore, reflection influenced work engagement indirectly through resilience (*β* = 0.34 × 0.31, *R^2^* = 0.011). Likewise, self-efficacy influenced work engagement indirectly through reflection and resilience (*β* = 0.28 × 0.43 + 0.28 × 0.34 × 0.31 = 0.14, *R*^2^ = 0.019).

## Discussion

The aim of the researchers was to examine the association between teacher self-efficacy, reflection, resilience, and work engagement among Chinese EFL teachers with the aim of expanding the research into teacher-related factors. The findings indicated that self-efficacy directly predicted work engagement, as confirmed by several prior studies (Skaalvik and Skaalvik, 2014; Burić and Macuka, 2018; Minghui et al., 2018; Han and Wang, 2021; Topchyan and Woehler, 2021). These studies found that teachers with higher self-efficacy tend to have a higher level of work engagement and are more persistent and diligent, with reduced levels of anxiety. This is in line with Bandura (1997) social cognitive theory, which suggests that individuals with high self-efficacy are motivated to perform better at work. EFL teachers who are confident in their skills and abilities to meet their students’ needs and run an effective course may become more motivated and invested in their teaching activities. According to Tschannen-Moran and Hoy (2001), teacher competence affects ambition, perception, and efficiency, and this was supported by Burić and Macuka (2018), who found a positive relationship between self-efficacy and work engagement. This result suggests that when teachers are confident in their ability to induce learning, they allocate more time and effort to their job and become more engaged in it.

The second finding of this study was that teachers’ level of reflection is positively associated with their level of engagement. Work engagement is defined as the opposite of burnout, therefore this finding is partially in line with several studies which indicated adverse link between teacher reflection and burnout (e.g., Cimermanová, 2013; Shirazizadeh and Moradkhani, 2018). Teacher reflection was negatively correlated with burnout, suggesting that reflective teachers are less probable to burnout, therefore they become more engaged in classroom. It can be contended that these two factors are interconnected in the sense that when a teacher constantly reflects his/her action, he/she becomes more involved in his/her work and achieves more favorable results. The opposite is also true, in that a highly involved teacher continuously reflects on his or her own practice in order to enhance its quality. Furthermore, it could be argued that teachers who demonstrate higher levels of reflection, are constantly thinking about their teaching practice and are thoroughly focused on enhancing their teaching standards. These instructors appreciate their jobs and are more deeply invested in them. As a matter of fact, they are better at managing and guiding both positive and negative emotions, and as a consequence, they can deal with stressful situations more efficiently. Put simply, instructors’ reflection can assist them to better control their feelings and feel more confident in the face of burnout. In other words, reflective teachers seem to be more dedicated and emotionally connected to their profession because they are fully engaged in thinking about their students and attempting to find solutions to issues they face. As a result, such teachers see classroom obstacles as catalysts for further learning and clarification of their classroom instruction (Uştuk and De Costa, 2021).

The last result of the current study showed a high relationship between teacher resilience and work engagement. The connection between teacher resilience and work engagement is smoothly demonstrated by the fact that teachers who can deal with the challenges of teaching get a lot out of their job. This, in turn, encourages instructors to become more engaged in their profession (Mansfield et al., 2016; Polat and İskender, 2018). This finding is partially in line with Polat and İskender (2018) discovery of an inverse interplay between teacher resilience and burnout. It is also asserted that teachers with higher stages of resilience experience less stress, resulting in a more potent feeling of belonging and greater faith in their abilities to live up to classroom expectations. In other words, teachers who exhibit greater levels of resilience are less tired and exhausted, have a greater level of job satisfaction, and are more able to maintain good collaboration with others. Instructors who are more resilient experience less occupational stress, which reduces the likelihood of burnout (Howard and Johnson, 2004). Furthermore, it was discovered that resilience could significantly predict EFL teachers’ teaching burnout. This finding is in line with the results of Richards et al. (2016)’s study that demonstrated a negative connection between teacher resilience and burnout. Another finding of this study was that teacher self-efficacy affected work engagement via the mediation of teacher resilience. Instructors with a higher sense of self-efficacy are much more dedicated to their instruction, develop a positive attitude, and are less likely to burnout. Instructors with reduced efficacy perceptions may maintain negative views of their instructional competences and the academic milieu, which increases the likelihood of feeling more psychological stress and detachment, as teacher self-efficacy deals with teachers’ views and beliefs of their own expertise in teaching and their efficiency in enhancing their students’ academic achievement.

Also, SEM results evinced that self-efficacy had an indirect effect on work engagement, which was mediated by teacher reflection and resilience. This finding suggests that the positive impact of teacher self-efficacy on work engagement is not only direct but also indirect. This is important because it highlights the role of teacher reflection and resilience as mediators in the relationship between teacher self-efficacy and work engagement. Concerning the mediating role of teacher reflection in the relation between work engagement and teacher efficacy, it can be argued that teachers with higher self-efficacy are more likely to enhance the overall learning environment in which they work, and thus are more likely to overcome obstacles and may have a higher job performance. It was discovered that teachers’ self-efficacy and reflection are positively associated. This is in line with the results of Cabaroglu (2014), who discovered a positive connection between teachers’ reflectivity and efficacy. Consequently, it can be asserted that highly reflective educators continuously consider their instructional practices in order to enhance its quality. This causes them to enjoy their work, become more efficient, and have faith in themselves. In addition, according to Fathi et al. (2021), self-efficacy and reflection can both be direct and negative predictors of burnout. As previously stated, work engagement is regarded as the opposite of burnout, therefore, positive correlations were thus not unexpected, lending credence to Schaufeli and Bakker (2004), Faskhodi and Siyyari (2018), and Ahmad et al. (2021) claim. There was also a negative relationship between teacher self-efficacy and burnout. This finding is consistent with the findings of several studies that highlight the crucial connection between these variables (Skaalvik and Skaalvik, 2014; Ventura et al., 2015). Such research has shown that teachers’ perspectives of their own qualities in handling teaching activities influence their job satisfaction and burnout. Teachers who have a greater sense of self-efficacy would be less likely to experience anxiety and burnout (Ventura et al., 2015). Thus, one could argue that because the EFL teachers in this study had more favorable attitudes about their own qualities in handling their classes, utilizing appropriate instructional strategies, and applying effective student engagement strategies, they felt less exhaustion.

Concerning the mediating role of teacher resilience, it was found teachers with greater self-efficacy perceptions are also more resilient, which in turn enhances their work engagement. These findings add to the growing body of literature on the interplay between various teacher individual and environmental factors that contribute to teacher wellbeing and job satisfaction. Self-efficacy, or the belief in one’s ability to successfully complete tasks, has been associated with positive outcomes in various domains, including education. Teachers with higher levels of self-efficacy tend to experience greater job satisfaction, higher levels of motivation, and lower levels of burnout (Bandura, 1977). The present finding highlights the importance of considering other factors, such as resilience, that may further amplify the impact of self-efficacy on work engagement (Luthar et al., 2000; Perera et al., 2018; Xie, 2021). Resilience, or the capacity to recover from setbacks and maintain wellbeing despite challenges, has been found to be a key factor in promoting positive outcomes for teachers (Van Wingerden and Poell, 2019). This finding that teacher resilience mediates the relationship between teacher self-efficacy and work engagement supports the idea that resilience acts as a protective factor that enhances the impact of self-efficacy on wellbeing (Tam et al., 2020).

Moreover, it was indicated that teacher reflection also had an indirect impact on work engagement, which was mediated by teacher resilience. This finding suggests that teacher resilience is a key factor in the relationship between teacher reflection and work engagement. The link between teacher reflection and teacher resilience has been empirically and theoretically supported in the literature (e.g., Beltman et al., 2011; Leroux and Théorêt, 2014). More particularly, teacher resilience enables teachers to better manage challenges and difficulties in their work environment, which in turn can enhance their ability to reflect on their practices and improve their work engagement. This highlights the importance of resilience as a mediator in the relationship between teacher reflection and work engagement, as well as its role in supporting teachers’ wellbeing and job satisfaction.

## Conclusion

To summarize the findings of this study, the connection among psychological constructs of teacher self-efficacy, resilience, reflection, and work engagement was revealed. The results of this study indicate that teacher self-efficacy, teacher reflection, and teacher resilience were significant direct predictors of work engagement. Furthermore, the study found that teacher self-efficacy had both direct and indirect effects on work engagement through the mediating roles of teacher reflection and resilience. Teacher reflection was also found to have an indirect effect on work engagement, which was mediated by teacher resilience. The constructs of self-efficacy, reflection, and resilience should receive more attention by researchers and educators as these constructs can affect teacher work engagement significantly. From the theoretical viewpoint, this study adds to the existing knowledge on work engagement by boldfacing the roles of self-efficacy, reflection, and resilience as predictors of work engagement among Chinese EFL teachers. By demonstrating the mediating effects of teacher reflection and resilience, the findings offered empirical support for the significant role that these constructs play in the development and maintenance of work engagement. However, because the numerous causes and qualifications associated with teacher resilience are less well-defined, more research is needed to carefully investigate the construct of resilience and to cultivate an all-encompassing foundation for teacher resilience that is both reasonable and empirically authenticated. Although it could be argued that the variables that enhance teacher resilience are primarily influenced by individuals’ experience and job status, it is also acknowledged that educator preparation programs can play an important role in enhancing teachers’ resilience. As Day and Gu (2014) correctly stated, improving teachers’ instructional quality and raising their students’ accomplishments and norms would necessitate the development of teachers’ resilience through early teacher preparation courses.

The results of this study might have significant implications for various stakeholders, including instructors, teaching staff, teacher training programs, school administrators, policymakers, and second language researchers. To support teachers’ work engagement, it is crucial for faculty members to educate both English teachers in coping with teaching challenges. Additionally, administrators should provide emotional, perceptual, and financial support, as well as ensure job stability, which are external factors that can impact teacher engagement. The outcomes can help EFL teachers understand the relationship between their self-efficacy beliefs, work engagement, and reflectivity. As a result, they can allocate more time and energy toward their profession and enhance their pedagogical skills through reflective practices, recognizing the impact of both intrapersonal and interpersonal factors on their job effectiveness. The findings highlight the importance of incorporating personality development in teacher training courses, in addition to practical teaching techniques. Teacher training programs can use these results to design and offer courses that cater to the emotional and psychological needs of EFL teachers and provide appropriate methods to increase their self-efficacy and work engagement. Professional development opportunities tailored to teachers’ job levels can enhance their skills and self-efficacy, reducing job stress and increasing job satisfaction. Enhancing teachers’ sense of self-efficacy leads to a higher commitment and engagement in teaching, resulting in a more fulfilled and satisfied profession. Language schools and institutions can contribute to improving their teachers’ self-efficacy by creating a supportive community, giving teachers autonomy, and fostering a sense of belonging. Bandura’s theory of self-efficacy highlights the importance of promoting both student and teacher self-efficacy, with school administrators playing a crucial role in supporting new teachers in their early professional years. This can be achieved through informal assistance and guidelines, as well as formal tutoring or peer-based support. Reducing the workload and increasing systematic emphasis and reflection on their teacher training and instructional role can also help newly qualified teachers avoid burnout and maintain their engagement in their work.

The outcomes of our study also have implications for instruction and the retention of teachers in the EFL educational system. Firstly, our findings suggest that fostering teachers’ self-efficacy, reflection, and resilience can contribute to increasing their work engagement. Therefore, teacher education programs and professional development initiatives should focus on improving these predictors of work engagement to enhance teacher effectiveness and satisfaction in the profession. Specifically, teacher education programs should integrate reflective practices and resilience-building strategies into their curriculum to provide future teachers with the necessary skills to cope with the challenges of the profession. Furthermore, as work engagement has been shown to be negatively associated with intention to leave the teaching profession (Høigaard et al., 2012), our study underscores the significance of promoting teacher work engagement as a means to retain teachers in the educational system. Improving teacher self-efficacy, reflection, and resilience can not only enhance work engagement but also reduce the likelihood of teachers leaving the profession due to burnout or low job satisfaction (Hong, 2012; Perera et al., 2018). Therefore, policymakers and school administrators should prioritize creating a supportive work environment that promotes these predictors of work engagement to improve teacher retention rates.

The current study has several limitations that should be acknowledged. One of the main limitations is that it relies only on self-reported data, which may impact the consistency and validity of the findings. In the future, researchers may choose to supplement self-reported data with qualitative methodologies such as semi-structured interviews or observations. Additionally, a mixed-methods approach could provide deeper insight into the relationship between the study variables. Moreover, the data was gathered only from EFL teachers in China, which may undermine the generalizability of the outcomes to other cultural contexts. Further research is needed to investigate if the findings can be extended to other cultures by collecting data from a diverse range of settings. Additionally, future studies could investigate the impact of socioeconomic factors, age, gender, and education level on the relationship between the study variables. Furthermore, as teachers’ attitudes can change over time, longitudinal research methods could be used to investigate and anticipate patterns of change in these instructor constructs.

## Data availability statement

The raw data supporting the conclusions of this article will be made available by the authors, without undue reservation. Requests to access these datasets should be directed to QH, 18409308@masu.edu.cn.

## Ethics statement

The studies involving human participants were reviewed and approved by the Department of Basic Education, Chongqing Creation Vocational College, Yongchuan 402160, Chongqing, China. The patients/participants provided their written informed consent to participate in this study.

## Author contributions

QH and LC were equally involved in designing the research, topic development, data collection, data analysis, writing drafts, and final editing. All authors contributed to the article and approved the submitted version.

## Funding

This paper was the research result of “Research on the Evaluation Dimension of Ideological and Political Teaching of Public Basic Courses in Higher Vocational Education” (No. 21SKGH393) sponsored by the Humanities and Social Sciences Project of Chongqing Municipal Education Commission, and “Research on the Evaluation System of Ideological and Political Teaching of Public English Courses in Higher Vocational Education” (No. 2021-GX-495) sponsored by the planning project of Chongqing Academy of Education Science.

## Conflict of interest

The authors declare that the research was conducted in the absence of any commercial or financial relationships that could be construed as a potential conflict of interest.

## Publisher’s note

All claims expressed in this article are solely those of the authors and do not necessarily represent those of their affiliated organizations, or those of the publisher, the editors and the reviewers. Any product that may be evaluated in this article, or claim that may be made by its manufacturer, is not guaranteed or endorsed by the publisher.
